# Spatial Access Priority Mapping (SAPM) with Fishers: A Quantitative GIS Method for Participatory Planning

**DOI:** 10.1371/journal.pone.0068424

**Published:** 2013-07-16

**Authors:** Katherine L. Yates, David S. Schoeman

**Affiliations:** 1 Environmental Science Research Institute, University of Ulster, Coleraine, United Kingdom; 2 Faculty of Science, Health, Education and Engineering, University of the Sunshine Coast, Maroochydore DC, Queensland, Australia; Bangor University, United Kingdom

## Abstract

Spatial management tools, such as marine spatial planning and marine protected areas, are playing an increasingly important role in attempts to improve marine management and accommodate conflicting needs. Robust data are needed to inform decisions among different planning options, and early inclusion of stakeholder involvement is widely regarded as vital for success. One of the biggest stakeholder groups, and the most likely to be adversely impacted by spatial restrictions, is the fishing community. In order to take their priorities into account, planners need to understand spatial variation in their perceived value of the sea. Here a readily accessible, novel method for quantitatively mapping fishers’ spatial access priorities is presented. Spatial access priority mapping, or SAPM, uses only basic functions of standard spreadsheet and GIS software. Unlike the use of remote-sensing data, SAPM actively engages fishers in participatory mapping, documenting rather than inferring their priorities. By so doing, SAPM also facilitates the gathering of other useful data, such as local ecological knowledge. The method was tested and validated in Northern Ireland, where over 100 fishers participated in a semi-structured questionnaire and mapping exercise. The response rate was excellent, 97%, demonstrating fishers’ willingness to be involved. The resultant maps are easily accessible and instantly informative, providing a very clear visual indication of which areas are most important for the fishers. The maps also provide quantitative data, which can be used to analyse the relative impact of different management options on the fishing industry and can be incorporated into planning software, such as MARXAN, to ensure that conservation goals can be met at minimum negative impact to the industry. This research shows how spatial access priority mapping can facilitate the early engagement of fishers and the ready incorporation of their priorities into the decision-making process in a transparent, quantitative way.

## Introduction

As the human population grows, pressure on the marine environment escalates and impacts of our unsustainable use can be seen in biodiversity loss, reduced water quality and declining fish stocks [1]–[6]. Efforts to improve management of the marine environment, and the resources within it, are becoming increasingly spatial, specifically through the development of marine spatial planning (MSP) and the designation of marine protected areas (MPAs). Whilst these management tools offer many potential benefits [7]–[15], too often these fail to be delivered [16], [17], or the planning process itself fails [18], [19].

One reason for the failure of MSP and MPAs is lack of adequate stakeholder involvement, even though this is often stated as vital for a successful planning process [20]. Incorporating stakeholders is important because it allows the planner to better appreciate the context of the plan and the potential impacts of different planning options. This greater appreciation provides the opportunity to develop more inclusive plans, which take into account the needs of all parties. By so doing, a planner should be able to both reduce conflict and help to develop a sense of stakeholder ownership over the project. Compliance with a chosen plan can be enhanced by this perception of ownership and inclusiveness [21]. Engaging with stakeholders provides the opportunity to explain the need for management changes, increasing understanding amongst stakeholders, which can again improve compliance [21]. Involving stakeholders can also facilitate generation of information that may not have otherwise been available, such as data on the distribution of species. This information can be incorporated into the decision-making process, which enhances planners’ abilities to find the most efficient solutions, thereby maximising benefits whilst minimising conflict and negative impacts. Stakeholder involvement, therefore, is vital not only to increase a project’s chances of success, but also to enhance the quality of decisions and reduce implementation cost [20], [22]–[25].

In the marine environment, one of the largest commercial stakeholder groups is the fishing community. They are also arguably the group that are most likely to suffer direct adverse economic and social impacts of spatial restrictions. For planners to be able to assess the impact of different options on the fishing community, they need spatial information on the relative importance of different parts of the sea. However reliable data for even the distribution of target species is all too often unavailable [26], with arguably even less known about the distribution of fishing effort, and practically nothing about priorities of the fishers themselves. To fill some of the gaps, researchers and managers have begun to involve fishers in participatory mapping studies, and the importance of such initiatives for effective management is increasingly accepted [27].

Participatory mapping projects have documented a diverse range of features, including: the locations of fishing grounds; the distribution of habitats and species; key management areas such as spawning grounds; and benthic and oceanographic features [26], [28]–[36]. These projects provide invaluable information for marine management. However, the maps are of limited use to a planner trying to take into account stakeholder priorities. First, these maps are frequently qualitative, making transparent quantitative analysis of the different options difficult. Second, even when quantitative data on stocks are available, they do not explicitly provide information on fishers’ access priorities: the distribution of resources does not necessarily relate directly or linearly to the importance of different areas to fishers or to the impact of restricting access to them. Nor should a planner assume to understand the ways in which other variables interact with the spatial distribution of the resource to determine the importance of an area to a fisher.

The development of vessel monitoring systems (VMS) attached to fishing boats has begun to provide very accurate, high-resolution quantitative data on the distribution of fishing effort [37]. VMS is very useful, but alone it still only provides information on where most fishing occurs, which is not necessarily the same as which parts are the most valuable to fishers. A more complete picture of the gross economic value can be obtained by combining VMS data with daily logbook entries, then mapping the catch value of different parts of the sea to the fishery [38].

In many situations, however, VMS data are still not available: at present, VMS are generally deployed only on larger vessels and in developed countries. Detailed landings data are not always collected, nor are daily logbook systems always in place. Indeed, even in developed countries such as the UK, the extent and spatial distribution of inshore fishing effort can be poorly known (pers. obs). When data are collected, they might still be inaccessible [39]. Thus, VMS-based methods are only possible sometimes, for some fisheries. Even then, resultant data infer the value of different areas to fishers, instead of documenting them. Alone, VMS data are not able to account for all the factors that might affect how important an area is for a fisher. Grounds closer to home, for example, could be more important than their gross value implies because of reduced costs required to access them in both economic (e.g. less fuel) and/or social (e.g. coming home each night) terms. Other areas might provide relatively low revenue, but still be very important because they are the only areas available at a given time of year, due to weather conditions; in such cases, maintaining access is necessary for maintaining a regular flow of income. Furthermore, VMS documents current spatial fishing patterns, which in many cases are already restricted by area-based quotas, by-laws, and existing protected areas. Therefore VMS may not actually represent fishers preferred fishing grounds. Finally, VMS methods do not facilitate the active involvement of the fishers; they thus neither meet the recognised and increasingly legislated need to involve stakeholders, nor provide the opportunity for stakeholders to feel part of the decision-making process.

Here a novel method, spatial access priority mapping (SAPM), is described and demonstrated for the Northern Irish fleet. SAPM requires no VMS, logbook or landings data, is suitable for single or multiple fisheries of any type, and can be performed using only basic features of standard spreadsheet and GIS software. It actively engages fishers, documenting rather than inferring their priorities, and produces quantitative maps that can be combined easily with other data if available and desired.

### Study Area – the Northern Ireland Context

Marine management in Northern Ireland faces many challenges. Recent European legislation, such as the Marine Strategy Framework Directive, is setting new standards for the environmental status of coastal waters. A Marine Bill is currently pending in the Northern Ireland Assembly that will provide the Department of the Environment (DOE) with new powers to develop marine spatial plans and to designate marine conservation zones (MCZs), a new type of marine protected area. The European Common Fisheries Policy, which has significant impact on the Northern Irish Fleet, is under review. The Northern Ireland Inshore Fisheries Strategy is also under review by the Fisheries Division of the Department for Agriculture and Rural Development (DARD). In addition, there is significant focus on the development of marine renewable energy in Northern Irish waters [40]–[42].

Whilst these developments bring many potential benefits for the Northern Ireland community, they will likely also bring spatial restrictions for fishers. In order to properly take fishers into account, and to make the most efficient plans possible, planners need to understand which parts of the sea are most important to fishers. However data are patchy at best. VMS is currently only in place for vessels 15 m and longer. Associated data have been processed to produce effort maps for some of their range (Irish Sea, but not the Clyde or other areas) [43]. Information on the inshore fishing fleet, which relies on smaller vessels, is limited, with no current monitoring of effort either in terms of magnitude or distribution. The distribution and stock levels of *Nephrops* are regularly monitored [44], and there are annual surveys for scallops [45] and ground fish [46]. There is, however, currently no information on the status of lobster or crab stocks, despite these resources supporting almost a third of the active vessels in the fleet. Access to the data that do exist is also restricted, sometimes even between Government Departments (pers. obs.).

Issues between the Northern Ireland Government Departments, particularly poor communication and co-operation between DARD and DOE, have caused problems for marine management in Northern Ireland in the past [47]. With DOE responsible for developing the spatial management plans, and DARD responsible for managing the fisheries that these plans are likely to impact, there is potential for further conflict. Thus, there is a real need for universally available data and a transparent way of incorporating the priorities of the fishers into the planning process.

**Figure 1:**
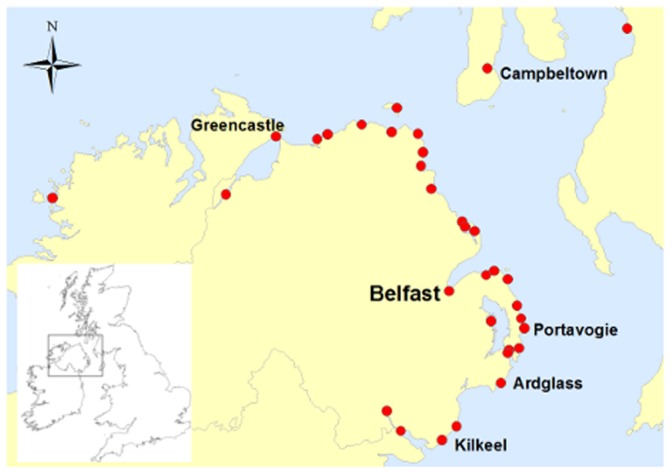
Map of the home ports from which commercially active, Northern Ireland registered vessels fish. Map of the home ports from which commercially active, Northern Ireland registered vessels fish Data obtained from the Department of Agriculture and Rural Development, Northern Ireland, 2012.

The majority of the Northern Irish fleet is based at three main ports: Kilkeel is the largest, followed by Portavogie and Ardglass. In addition, there are over twenty minor ports at which small numbers of pot boats are based (Figure 1). A few Northern Ireland registered boats also fish out of ports in neighbouring countries, mainly Greencastle in the Republic of Ireland. There are 367 vessels registered in the Northern Ireland fleet, 224 of which are officially recorded as active (DARD, pers.comm). The majority of those 224 vessels are under 15 m (127, 57%) and would thus not be covered by current VMS data.

The most important fishery in terms of revenue and employment is *Nephrops*, with 99 of the 224 vessels focusing virtually exclusively on *Nephrops*, and a further 11 mixed-fishery vessels having some *Nephrops* interest (DARD, pers.comm). Pot-fishing accounts for the second highest number of vessels, with 69 of the 224 registered as exclusively potting, and 8 mixed-fishery vessels having potting interest (DARD, pers.comm). There are two main Fish Producer Organisations (FPOs): the Anglo-Northern Irish Fish Producer Organisation (ANIFPO); and the Northern Ireland Fish Producer organisation (NIFPO). There are two pot fishers associations, the North Coast Lobster Fishermen’s Association and the North-East Lobster Fishermen’s Co-Operative, and a Scallop Fishermen’s Association. All of these representative bodies reported attempting to engage with the Government Departments to improve management.

## Methods

### Interviews

Research was approved by the School of Environmental Sciences Ethics Committee, University of Ulster, Coleraine. All interviews were conducted in accordance with ethics procedures, and research plans, including the consent procedure, were submitted and approved prior to the initiation of the project, in accordance with School and University policy. Prior to each interview, information sheets detailing the research were provided to fishers and discussed, and then verbal consent was obtained from participants. Written consent was not sought, partly to maintain the informal setting that assisted with the response rate, but mainly because the respondents were not required to give their names during the interview, thus requiring a signature (a form of identification) was considered inappropriate. Verbal consent was recorded in the response form. All interviews were conducted by the lead author, ensuring that the procedure was uniform for all respondents, and all individual responses were recorded in a confidential database. The pre-interview information sheet and questionnaire are available in Files S1 & S2.

Interviews consisted of two parts: a semi-structured questionnaire; and a mapping exercise. Each interview lasted between 30 minutes and two hours. The vast majority of fishers interviewed (>90%) were approached directly at ports. A number of other fishers responded to flyers that had been distributed by mail. All respondents were skippers or vessel owners. With the exception of three interviews conducted in fisher’s homes (2.9%), all interviews were conducted in ports (97.1%): on vessels, at the quayside, in a cafe, or in the ANIFPO or NIFPO offices.

During the mapping exercise, fishers were provided with both paper admiralty charts and digitised admiralty charts within a GIS. Fishers indicated their priority areas (as polygons) directly onto the digitised charts. They were encouraged to identify as many or as few areas as they liked, and to cover as much area as they wanted. However, each respondent was advised that the greater the total area, the lower the priority that could be assigned to each unit of area; thus they were encouraged to be specific. Those fishers active in multiple fisheries were asked to indicate the target species of each area. They were given the option of assigning areas different levels of importance and/or indicating how much of the year they spent in each area, which was then converted into a percentage. This then became the importance value, such that an area that was fished for a fifth of the year received an importance value of 20. On completion of the mapping exercise, respondents were encouraged to review their results and to make changes as desired.

### Generating the Maps

**Figure 2:**
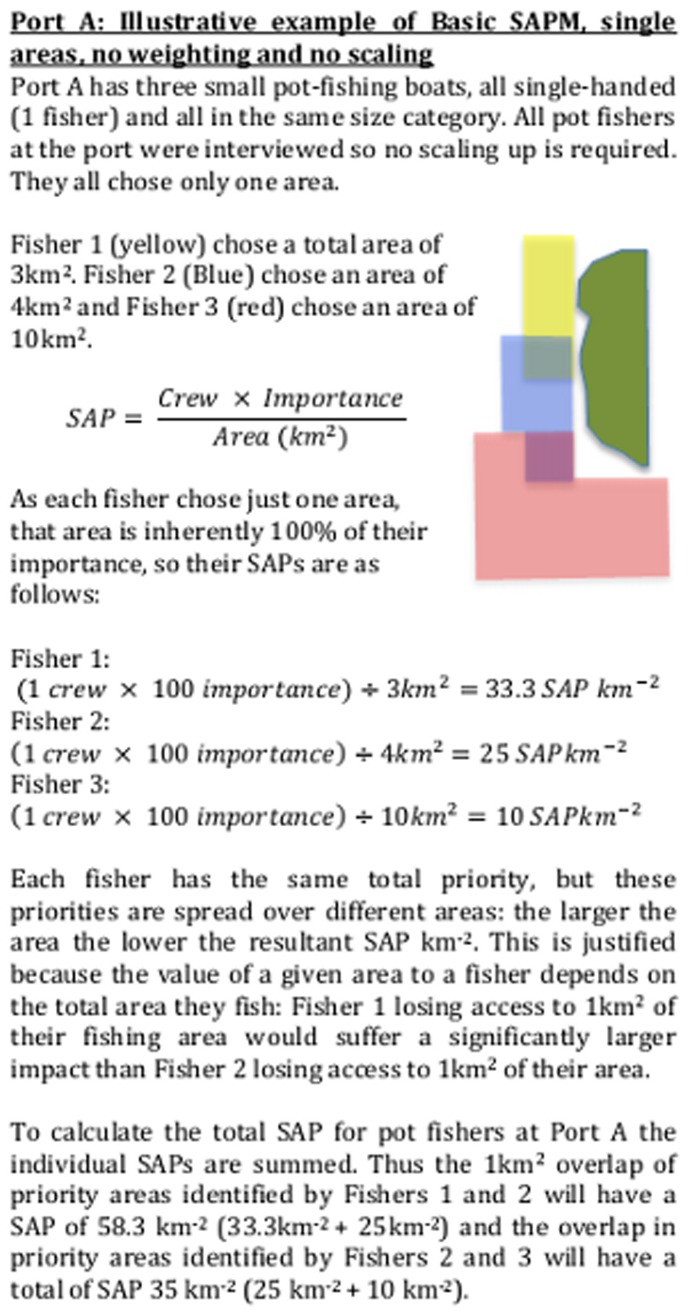
Illustrative example of basic SAPM. Illustrative example of basic SAPM, no weighting or scaling involved.

**Figure 3:**
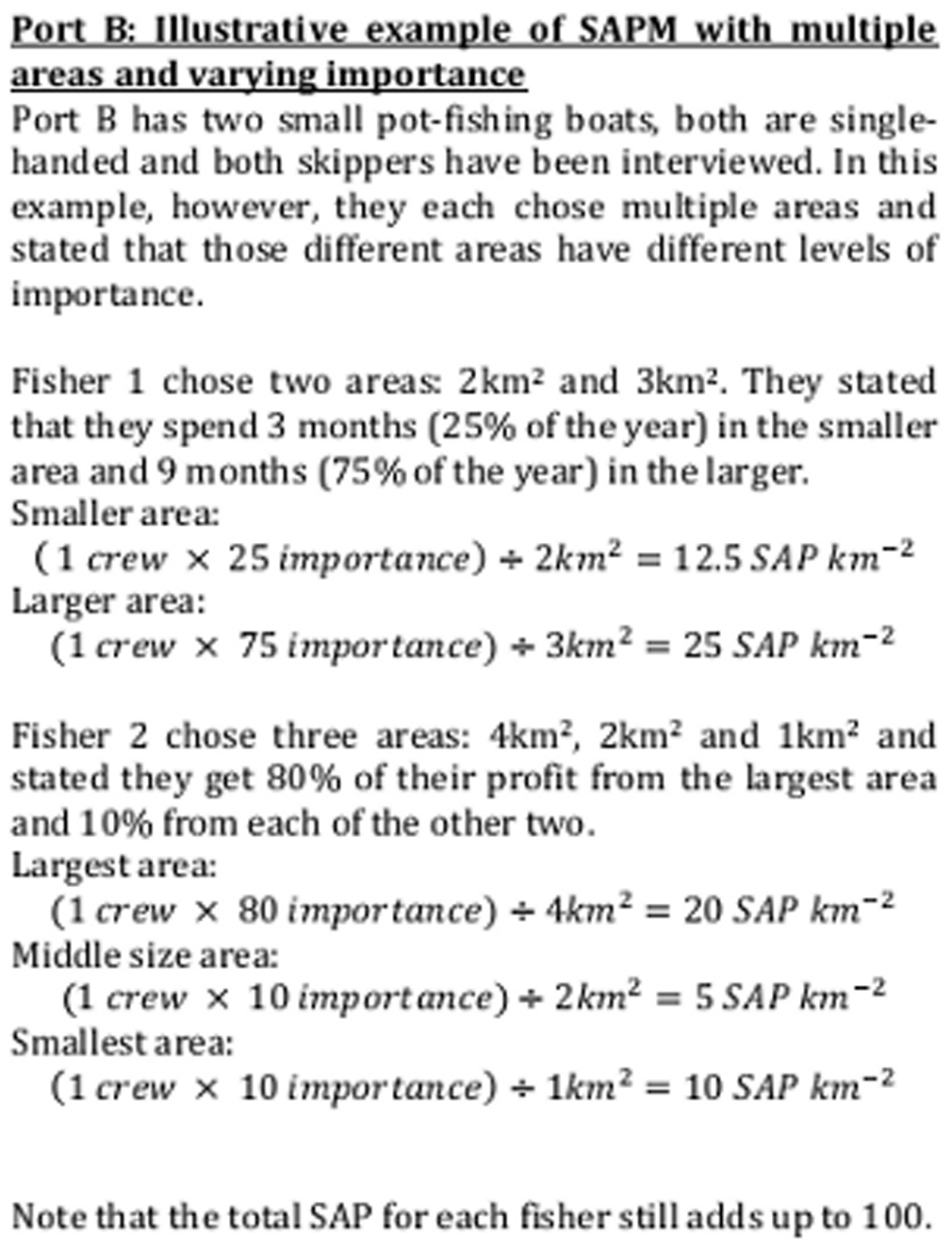
Illustrative example of SAPM with multiple areas and varying importance. Illustrative example of SAPM with multiple areas and varying importance.

The priority (SAP km^−2^) of each of the areas indicated by a particular respondent was calculated by multiplying the number of full-time crew on the associated boat by the importance value, and then dividing by the number of square kilometres the area covered (Figures 2 & 3 show illustrative examples). Part-time or seasonal crew were assigned a *pro rata* proportion of a full-time member’s weighting, so that a boat that reported four full-time staff throughout the year and one extra full-time staff for six months of the year was recorded as having a total of 4.5 crew. Each individual (full time) fisher has a total SAP of 100, such that a boat with a crew of 4.5 has a total SAP of 450 associated with it, and an area that has an SAP of 10 km^−2^ represents a tenth of a fisher per km^2^.

**Figure 4:**
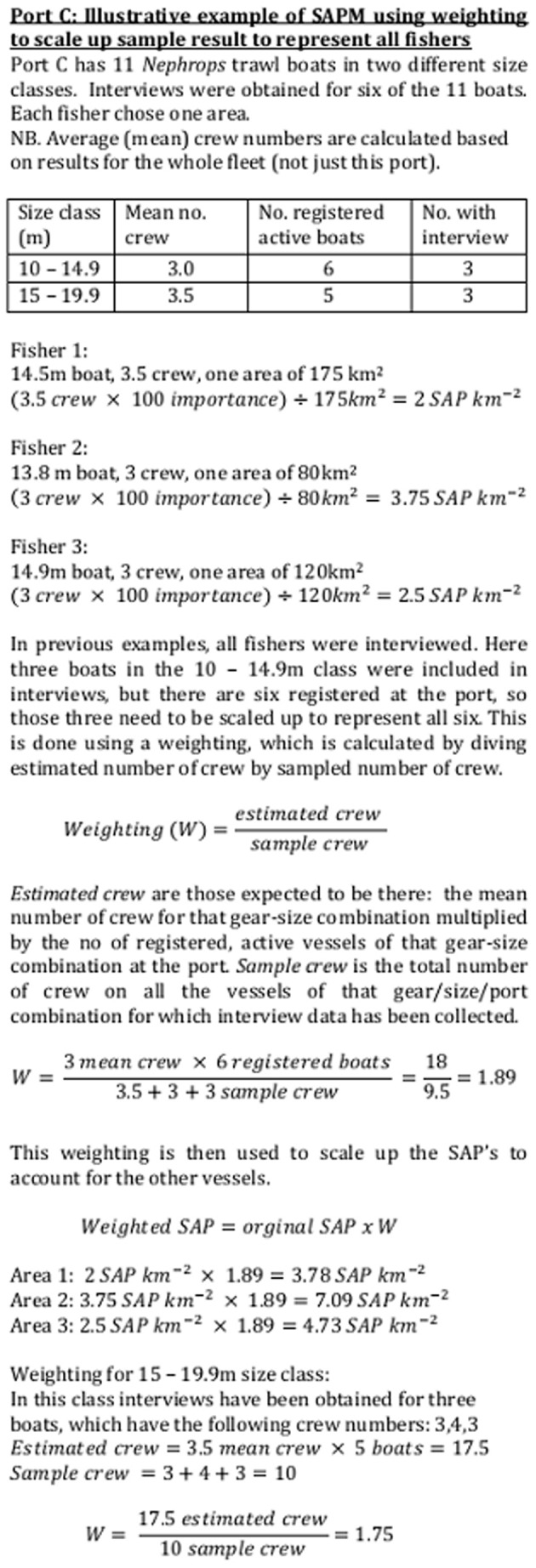
Illustrative example of SAPM using weighting to scale up the sample to represent the population. Illustrative example of SAPM using weighting to scale up the sample (interviewed fishers) to represent the population (whole fleet).

**Figure 5:**
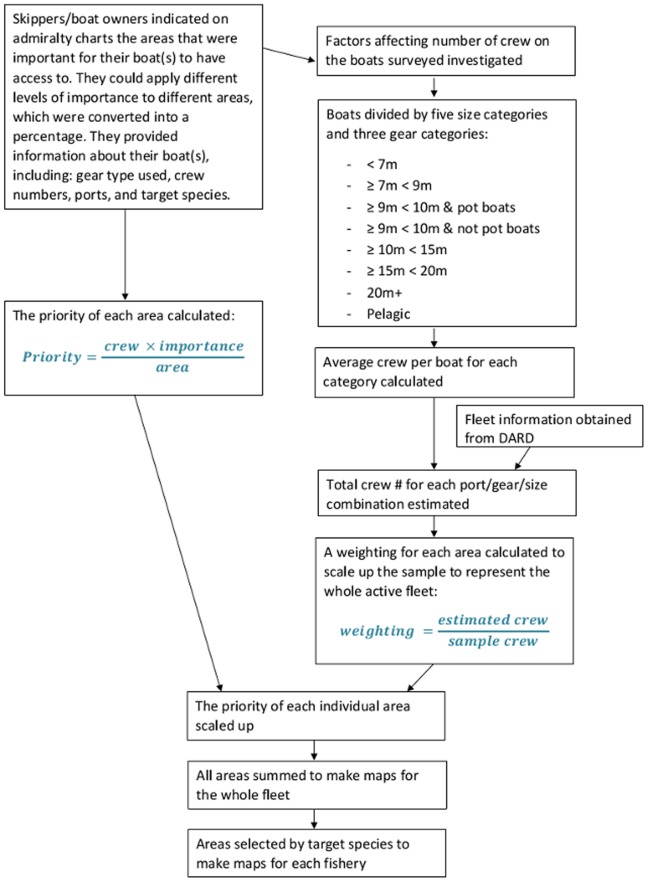
Flow diagram of the Spatial Access Priority Mapping Method. Flow diagram of the Spatial Access Priority Mapping Method use to map the access priorities of the Northern Ireland fleet in 2012. DARD stands for the Department of Agriculture and Rural Development, Northern Ireland.

In order to create maps that represented the whole fleet, the results obtained from the individual respondents need to be scaled up (Figure 4 shows an illustrative example). Based on initial examination of data and discussions with the fishers, vessels were divided into six size categories. All vessels under 9 m were pot boats. With the exception of two specialist offshore crab boats, no vessels over 10 m fished pots. In the ≥9 m <10 m category there was a mix of pot boats and small dredge or trawl boats. Dredge and trawl boats of similar size generally have very similar numbers of crew, but pot boats of the same size tend to have fewer crew and are often single handed. A two-sided t-test demonstrated that there were significantly fewer crew on pot boats between 9 and 10 m than other gear types (t = 3.05, df = 20, p = 0.007). Consequently, this size category was further divided by gear: pots or not pots. Data was obtained for only one pelagic vessel, of which there are three in Northern Ireland; however, an additional category was assigned for pelagic vessels as they have much higher crew numbers (8–10) than any other type. Thus vessels were divided into a total of eight categories (Figure 5) and the average number of crew per vessel for each of these categories was calculated using the sample data.

A list of vessels at each port was provided by the fisheries division of DARD, along with the length and registered gear type of each vessel. The list showed 224 active registered vessels, but during the research a number of inaccuracies were found with this list and it was updated to include additional boats: 14 that were originally listed as ‘no recorded fish effort this year’, and 8 that were not listed. The final vessel list contained 246 vessels and was broken down by port, gear type and length. Using this and the calculated average crew per vessel, an estimate was made of the total number of fishers on all active vessels by each port, gear and length combination.

**Figure 6:**
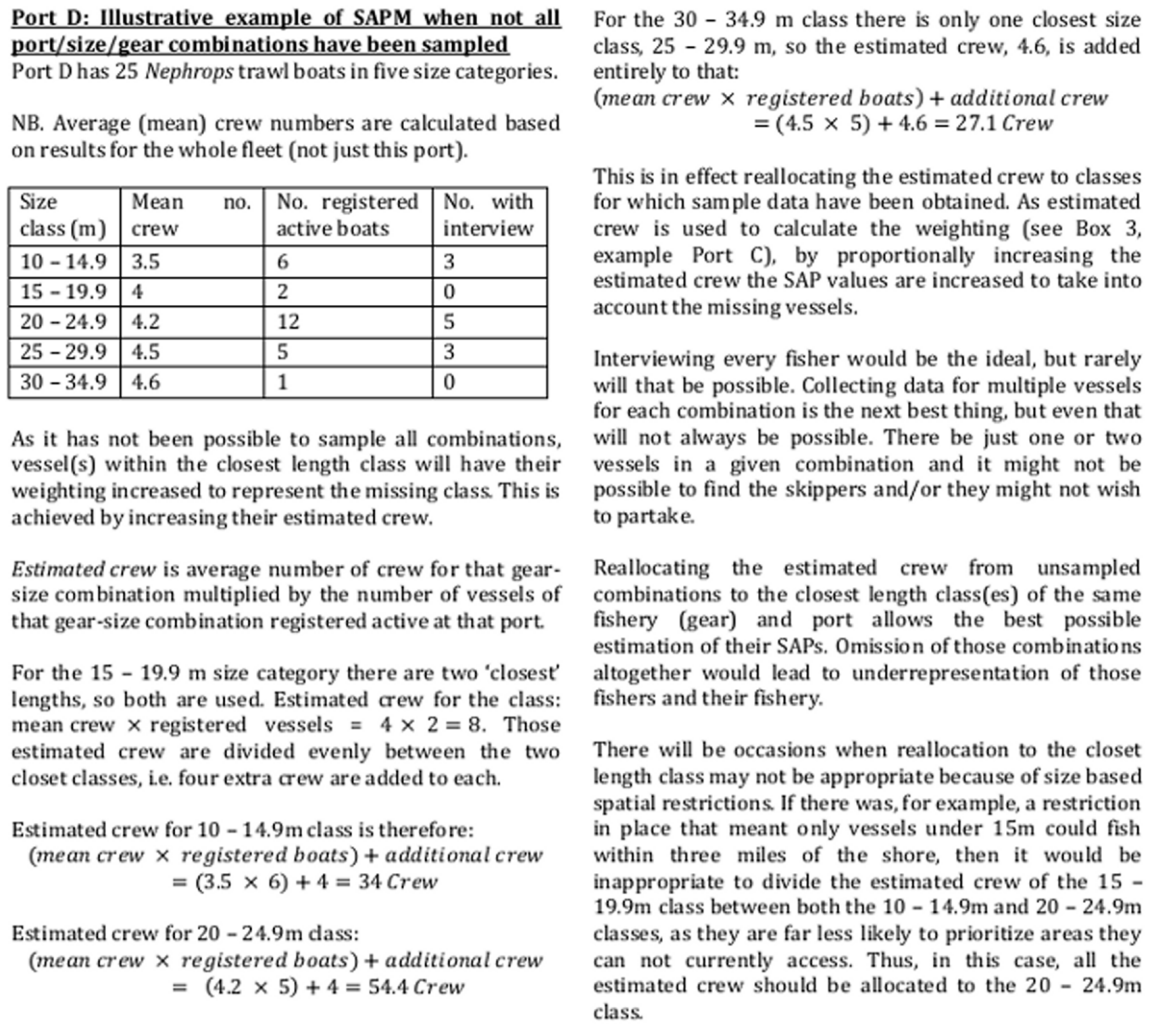
Illustrative example of SAPM when not all gear-port-size combinations have been sampled. Illustrative example of SAPM when not all gear-port-size combinations have been sampled.

In each length/gear/port combination, the estimated number of crew was divided by the actual number of crew sampled in order to create a final weighting scheme, which was used to upscale the maps generated by respondents to represent the whole active fleet (Figure 4). Where no skipper of a vessel with a particular gear-length-port combination had been interviewed, responses from skippers at that port, using that gear, whose vessels most closely matched the required length, were upweighted appropriately (Figure 6 shows an illustrative example). On the very few occasions (3) where no skipper of vessels of that gear type and port had been interviewed, appropriate response(s) from the nearest port were used.

Priorities for each individual area were then multiplied by their corresponding weights to obtain a final weighted priority score. Resulting shape files were rasterised, at a resolution of 1 km^2^, and all raster layers were combined using the raster calculator to construct the overall access priority map for the Northern Ireland fleet. Access priority maps for different sectors of the fleet were generated by selecting only the layers that related to that sector. To demonstrate the simplicity of the approach, all calculations were performed using tables and basic cell formulae in an Excel spreadsheet. Other freely available software such as R could be used as an alternative, making the analysis much quicker without changing the method. All spatial analysis was conducted in ArcMap 9.3.

### Validation

In order to assess whether the sample of respondents was representative of the whole fleet, the three main characteristics of vessels were compared. A two-sided t-test was conducted to ascertain whether there was any significant difference in the vessel length distribution between the sample and the population. Chi-squared tests were used to test for any significant difference between the sample and the population in terms of the frequencies of gear types and registered ports. The smallest ports were grouped geographically to make this possible.

SAP maps from individual fishers within a given length-gear-port combination were overlaid to inspect for consistency of responses and verify the validity of up-scaling to represent all vessels. Variation is to be expected between different combinations, and even some minimal variation within combinations. However if the responses within a combination were very different up-scaling would not have been appropriate.

VMS-based effort maps (2009 and 2010) for boats 15 m and longer were provided by AFBI and were used to visually validate the areas indicated by those boats. It was not assumed that VMS is always an accurate representation of spatial access priorities. Indeed, as previously mentioned, an area may be fished for only a short time but may actually have a high value because of the quality of the catch or because it is the only site available at that time of year. Nevertheless, because it would be expected that all areas fished would have some priority associated with them, a visual inspection of the two sets of data provided a validity check for overall coverage. It would also be expected that a similar overall pattern would be evident.

Copies of the amalgamated maps for the whole fleet and for the four main fisheries, with the associated report [48], were made available to all members of the Northern Ireland fishing fleet to review. Digital copies were provided to the POs, fishers’ associations and co-operatives, for distribution to their members. Hard copies were provided to the harbour offices of the POs and to harbour cafes, where they were made available for fishers to review. In addition, individuals were able to download digital copies of maps and reports from the University of Ulster website, the availability of which was advertised in the national fishing press. Feedback was provided in a number of ways: POs and associations solicited and passed on responses from members, some fishers responded using forms and prepaid envelopes that were provided along with the hard copies sent to POs and cafes, a number of individuals submitted responses by email, and others made direct contact with the lead author to discuss the results.

## Results

**Figure 7:**
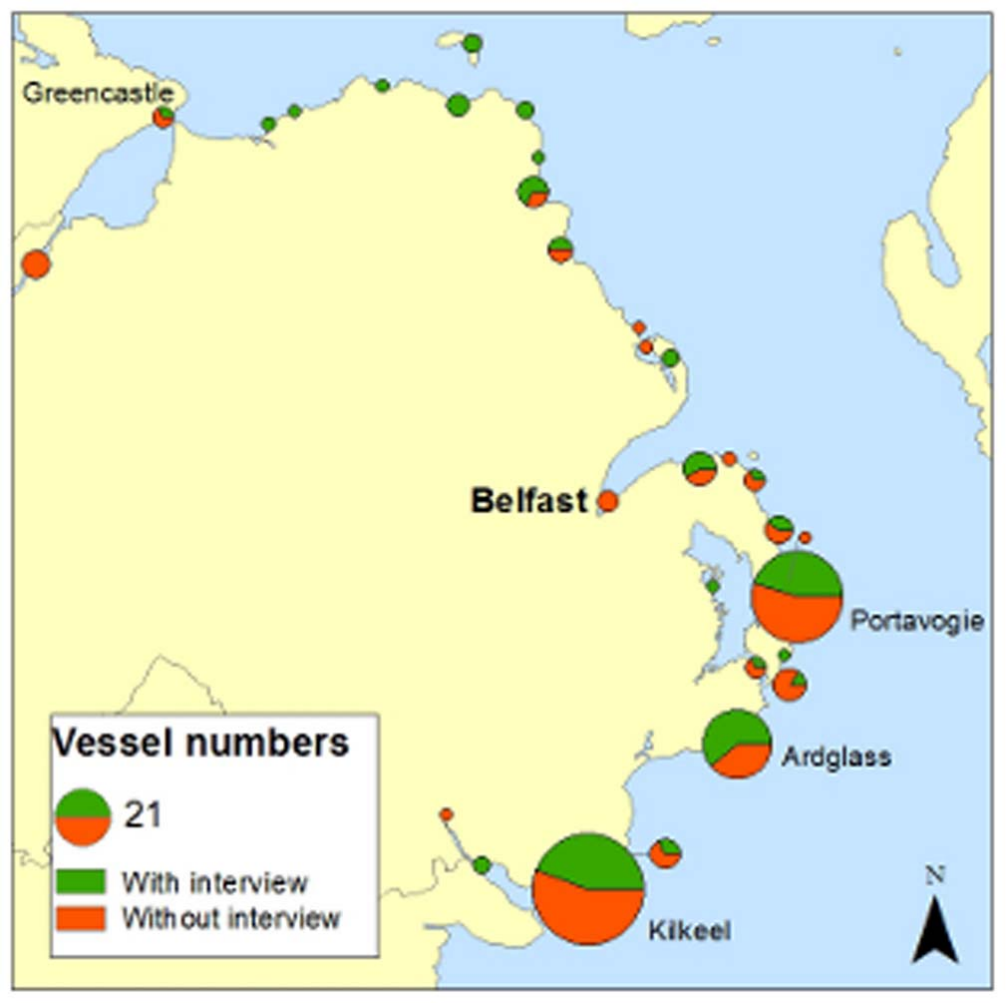
The proportion of vessels for which interviews were obtained or not obtained. Map of the Northern Irish ports, plus Greencastle, showing the proportion of vessels for which interviews were obtain for against those for which they were not. The size of the symbol for each port represents the number of commercially active, Northern Ireland fishing vessels registered there.

Interviews were conducted in 20 different ports. The response rate (97%) was excellent. Fishers were very willing, often keen, to participate once the project was explained. Indeed, many expressed concern that those making the decisions understood neither the industry nor the fishers’ needs, and were glad to have an avenue to express their opinions. In total, 103 individual skippers and owners of active vessels were interviewed. Only three skippers declined, two saying they were too busy and the other refusing to engage with the process. The respondents skippered and/or owned 118 vessels, representing 48% of the active fleet (updated list), and those vessels fish out of 25 different ports (Figure 7).

Overall, the sample is considered to be an accurate representation of the fleet (Tables 1& 2, Figure 7). The two-sided t-test on vessel length showed no significant difference between the sample and the population (t = 0.06, df = 334, p = 0.995). Chi-squared tests also showed no significant dependence between sample and fleet coverage either in terms of the registered port (X^2^ = 18.232, df = 19, p = 0.507), or the type of gear used (X^2^ = 3.095, df = 9, p = 0.96).

**Table 1:** Comparision of the number of registered active Northern Ireland fishing vessels and the number with interviews, by size class.

Size, in meters	Number active	Number with interviews	% with interview
under 7	48	21	43.8
7 to <9	17	10	58.8
9 to <10	49	22	44.9
10 to <15	32	19	59.4
15 to <20	67	35	52.2
20 to <30	29	9	31.0
30+	4	2	50.0
**Total**	**246**	**118**	**47.9**

The total number of registered active Northern Ireland fishing vessels and the number with interviews, by size class. Fleet information supplied by the Department of Agriculture and Rural Development, Northern Ireland, and update during the research.

**Table 2:** Comparision of the number of registered active Northern Ireland fishing vessels and the number with interviews, by gear type.

Gear	Number active	Number with interview	% with interview
Dredge	5	4	80.0
Dredge & Nephrops trawl	23	13	56.5
Gill Net	13	3	23.1
Hand Fishing	1	0	0.0
Nephrops & Whitefish trawl	5	2	40.0
Nephrops trawl	104	49	47.1
Pelagic trawl	3	1	33.3
Nephrops trawl & Gill net	1	0	0.0
Pots	80	42	52.5
Pots & Nephrops trawl	4	1	25.0
Pots, Dredge & Nephrops trawl	2	2	100.0
Scallop Dredge	2	1	50.0
Seine netting	1	0	0.0
Pots & Dredge	2	0	0.0
**Total**	**246**	**118**	**47.9**

The total number of registered active Northern Ireland fishing vessels and the number with interviews, by each gear type. Fleet information supplied by the Department of Agriculture and Rural Development, Northern Ireland, and update during the research. Please note that many scallop dredgers are registered as dredge or dredge and nephrops.

**Figure 8:**
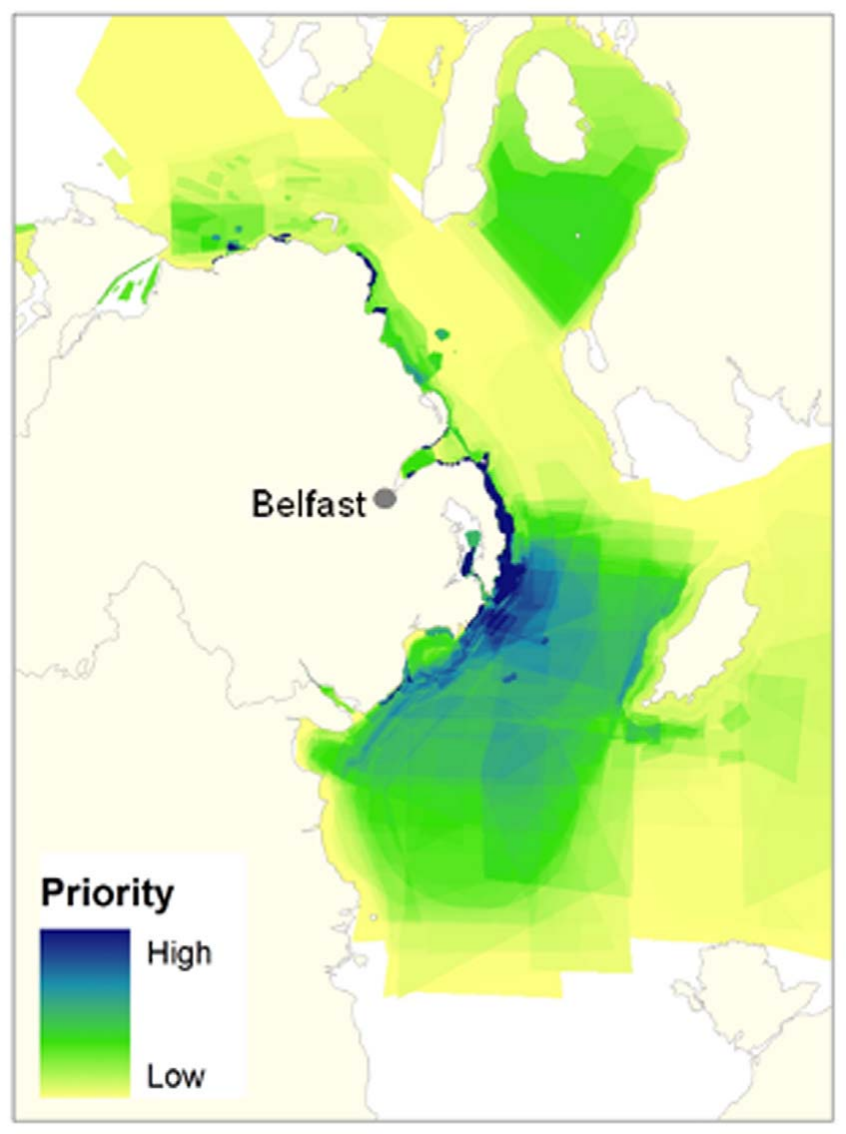
Map of the spatial access priorities of the Northern Irish fishing fleet. Map of the spatial access priorities of the Northern Irish fishing fleet generated from data obtained from 103 interviews with skippers and boat owners in 2012. It shows the main areas used by the fleet. There are a number vessels that have a much greater range than this. All areas were included in mapping process; however, for the purpose of maintaining an appropriate scale for visual display, they have not been shown.

**Figure 9:**
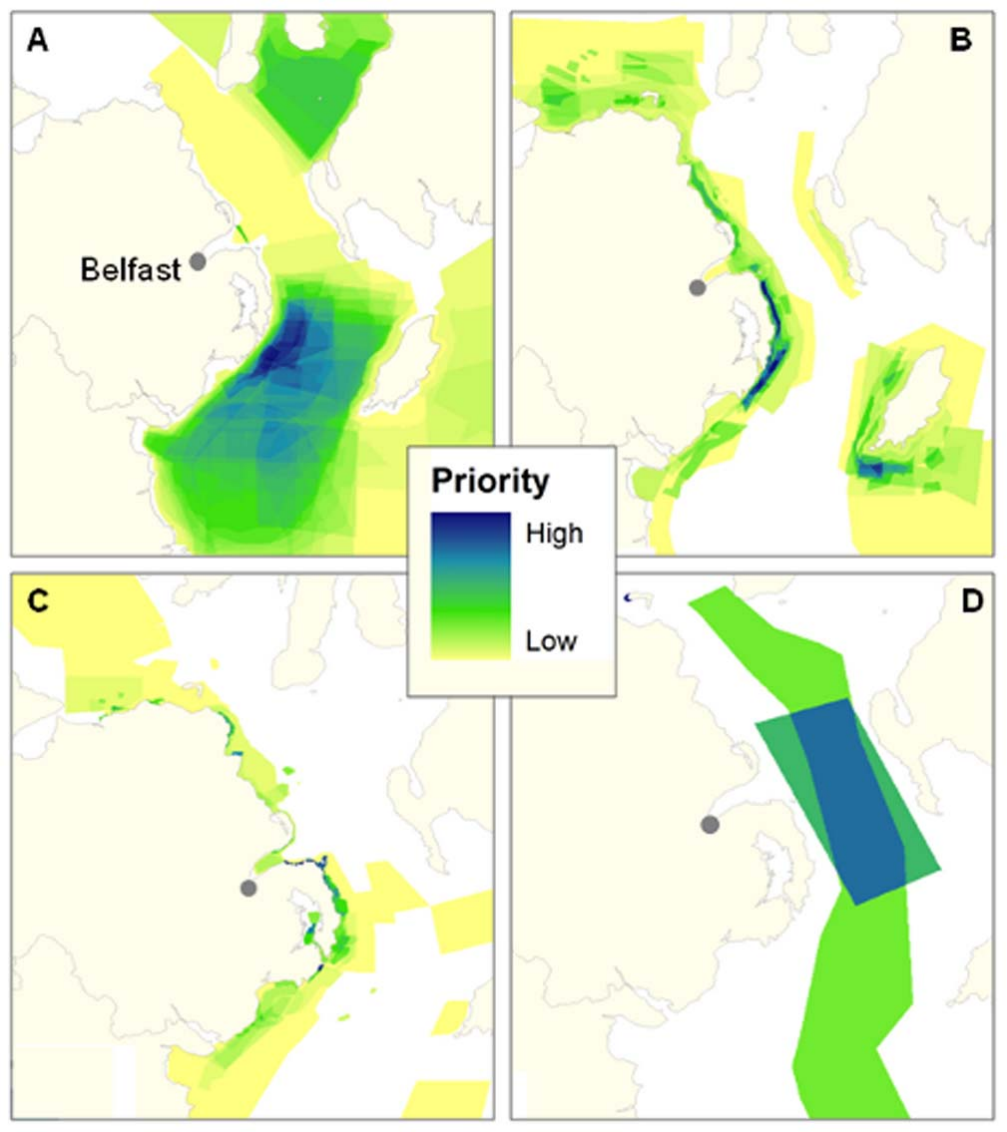
Maps of the spatial access priorities of the four main fisheries within the Northern Irish fishing fleet. Maps of the spatial access priorities of the four main fisheries within the Northern Irish fishing fleet: A) Nephrops, B) Pot fishing, C) Scallops and D) White fish. Maps were generated using data obtained from interviews with a total of 103 Skippers and boat owners in 2012. They show the main areas used by the fleet. There are a small minority of Nephrops vessels that have a greater range than this. All areas were included in mapping process; however, for the purpose of maintaining an appropriate scale for visual display, they have not been shown.

The map for the whole fleet produced by SAPM (Figure 8) shows the spatial heterogeneity in access priority and clearly highlights areas whose closure would likely cause greatest contention with the fleet. Unsurprisingly, results show that spatial priorities vary greatly between different fisheries (Figure 9) and, as closures are frequently only for certain types of gear, this can provide valuable input for planners and managers deciding what activities to restrict and where.

**Figure 10:**
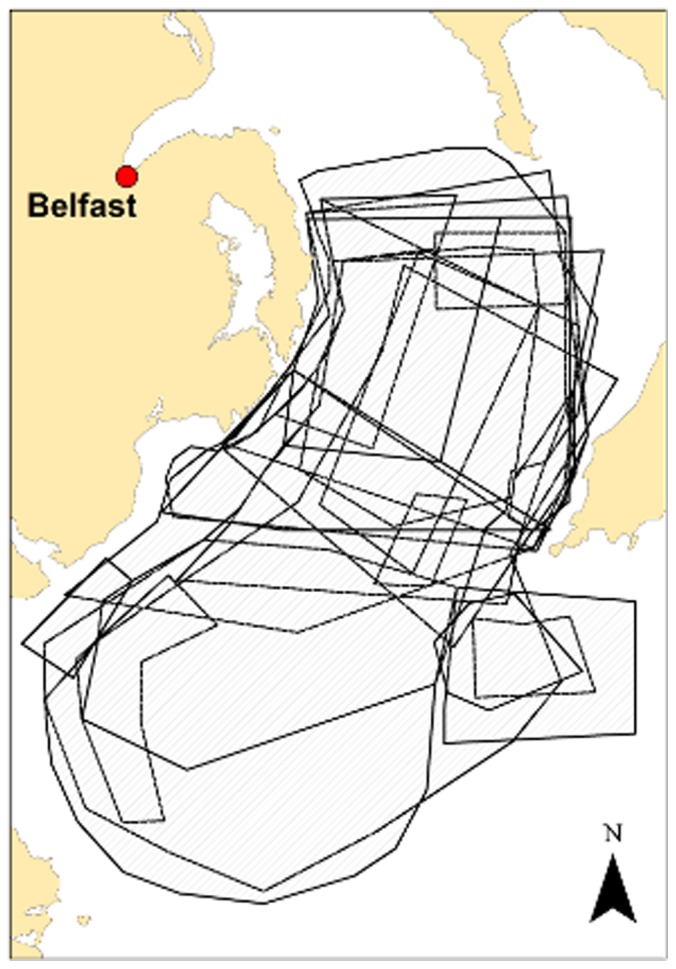
Spatial overlap of fishers’ access priority areas within a given gear-length-port combination. Map of the access priority areas chosen by nine *Nephrops* fishers, from Northern Ireland. The nine fishers’ vessels were within a given port-length combination and the map shows the spatial overlap in their responses.

Comparisons among results from individual respondents showed a high degree of spatial overlap within a given length-gear-port combination: Figure 10 shows an example from a combination within the *Nephrops* fishery. Overlap was high across all of the three main ports for the *Nephrops* fishery, within each size category. Between size categories, overlap reduced, with smaller vessels having a corresponding reduced range from port. Overlap was also high across the three main ports for the scallop fishery (dredge boats). However, overlap across ports for pot-fishing was very low. This is unsurprising, as the small vessels generally have a very restricted range and fishers can be territorial between and sometimes even within ports. This stresses the importance of obtaining good spatial representation of the pot-fishing community, which can be difficult when small ports have just one or two fishers operating from them. In this study, the involvement of the Lobster Associations was invaluable in ensuring good coverage.

**Figure 11:**
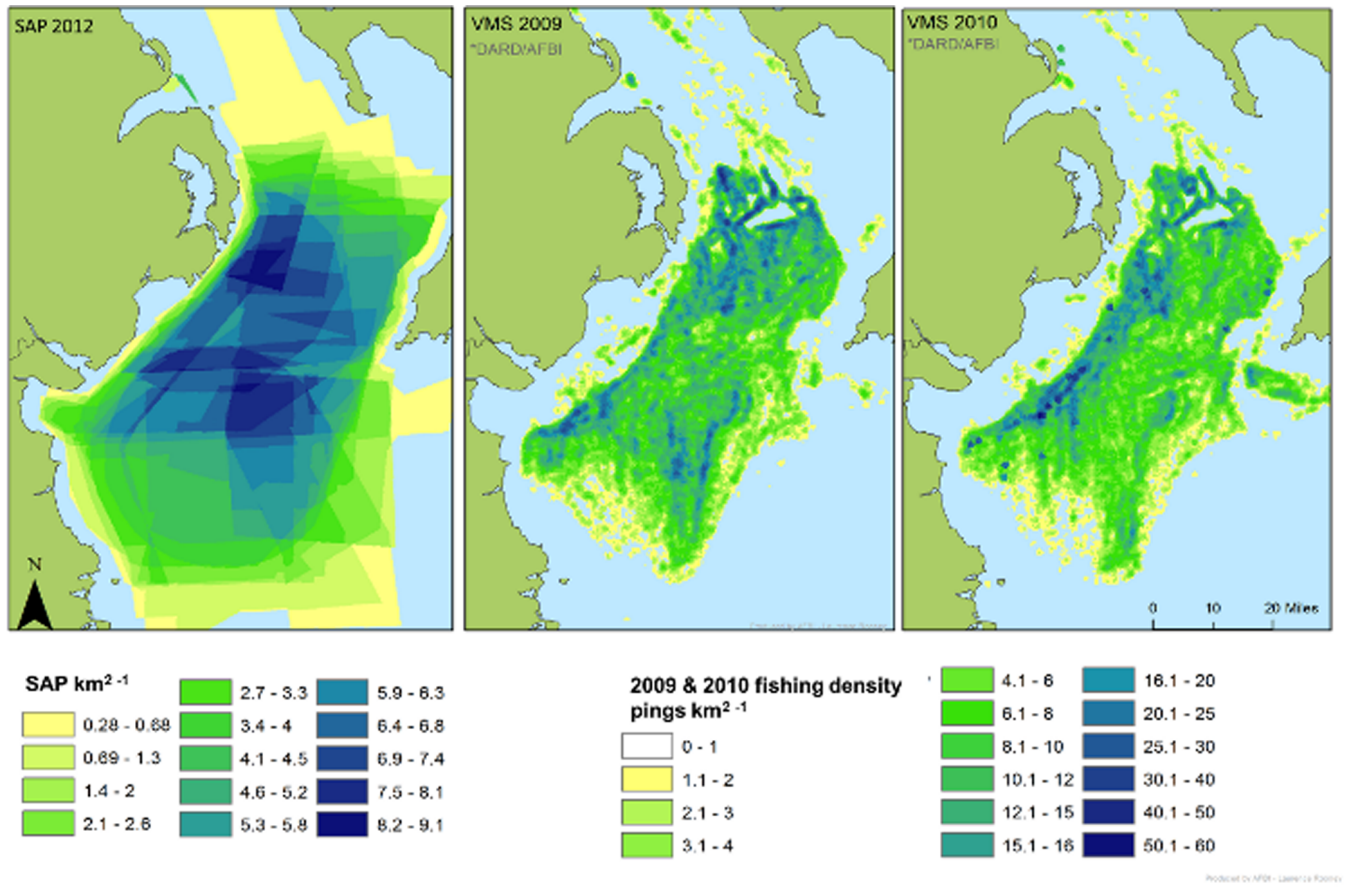
Spatial access priority map and VMS effort maps for the over 15 m *Nephrops* fleet. The spatial access priority map (2012) and the VMS effort maps (2009 and 2010) for over 15 m *Nephrops* fleet. VMS maps supplied by Laurence Rooney at AFBI. Processed VMS data were not available for all areas delineated by the fishers so this figure compares only main fishing areas where it was.

Overall, visual congruency between the access priority map for the over 15 m fleet and the VMS effort map is high, with all areas fished by the fleet being covered on the access priority map (Figure 11). The access priority map covers a larger total area than the VMS, which is unsurprising, and is most likely a combination of the inherently higher accuracy of the VMS and the wider scope of values that the access priority map captures.

Feedback on the initial report and maps was provided by 21 active skippers/owners, a 9% response rate, as well as by four retired fishers. Active skippers/owners represented all of the main fisheries and comprised: 8 trawl fishers; 9 pot fishers; 1 dredge/scallop fisher; and 3 fishers who targeted multiple fisheries. The response was very positive, with the result perceived by all respondents to be a good reflection of the fleet’s priorities. However, many fishers expressed concern that Government Departments would not use fishers’ inputs in forthcoming plans.

### Data Sharing

An important aspect of this highly applied research was to provide data to feed into impending management changes. Once the maps, and associated report, had been reviewed by the fishing community and their comments had been added, it was formally presented to a group of representatives from the relevant Northern Ireland Government Departments: DARD, AFBI, DOE, the Northern Ireland Environment Agency and the Department of Enterprise, Trade and Investment (Marine Renewable Energy Division). In addition to the digital and hard copies of the report, they were also provided with the raster data layers for the five maps (the whole fleet and the four main component fisheries) for use in their GISs. All agencies stated intentions to use these data in forthcoming marine spatial planning and MCZ-designation activities.

The report and data layers were also provided to the UK Marine Management Organisation, Natural England, the UK Joint Nature and Conservation Committee, and the Council for Conservation and the Countryside (Northern Ireland). The report is freely available to download [47], and interested parties can request the data layers from the lead author. Data layers will be deposited in the UK data archive in 2014. Raw data and all information relating to individual fishers are confidential and will not be made available.

## Discussion

More than 100 skippers and owners of Northern Ireland registered vessels were interviewed about their spatial access priorities: their boats represent almost half the active Northern Irish fleet. Data from these interviews was used to generate spatial access priority maps for both the whole fleet and for each of the main fisheries. These maps provide quantitative data on which parts of the sea are most important to the fishers, and they can be used to maximise the efficiency of protected area site selection and to minimise potential conflicts between conservation needs and those of the fishing industry. The maps can also be used to inform marine spatial plans, highlight areas of tension between fisheries and contribute to fisheries management plans.

This research demonstrates the willingness of Northern Ireland fishers to be involved in both research and management planning. The response rate was excellent, despite early concerns by many involved in local marine management that it would be difficult to get the fishers to participate. In fact, fishers were extremely forthcoming with a range of information and many were pleased to have the opportunity to “have their say”. The nature of the project may have been a factor in this. First, it was independent research, and thus it bypassed any historical animosity between the Government Departments and the fishers. Second, the research sought explicitly to map fishers’ access priorities so that these could be taken into account in future planning processes; it was therefore in fishers’ interests to take part. Third, the method was easy to explain to respondents: it was simple, intuitive and representative. This latter characteristic not only increases the chance of participation but also, increases the reliability of fishers’ responses because they are far more likely to genuinely understand what is expected of them. Probably the main factor, however, was that the research went to them: interviews took place on fishers’ boats, at the quayside, or in the cafes they frequent. This allowed the fishers to participate at a time and in a space that suited them, and it greatly reduced both the actual and opportunity costs of their participation. Indeed, many fishers complained that in order to participate they would normally have had to travel to meetings held in Belfast, at their own expense, and lose a day’s fishing. That situation is not unique to Northern Ireland [31] and can lead to poor and/or skewed representation, with only certain fishers able or willing to contribute.

The response rate for the review of the results was much lower than for the face-to-face interviews. This is not surprising: response rates to distance surveys are normally much lower than face to face interviews [49], because in face-to-face interviews the researcher has the opportunity to explain the research and the value of the respondents’ involvement. A survey discussing fishing revenue and expense data, distributed to 284 vessel owner/operators in Seattle, for example, had just 34 surveys returned, a 12% response rate [50]. It is also important to bear in mind that this response rate is artificially low because it is calculated by dividing the 21 respondents by all active skippers (246), when in fact it is unknown how many of those skippers were actually aware of the research, the review period or their opportunity to respond. The responses received also suggest that the lower response rate may in part reflect a general agreement with the results, combined with cynicism over the incorporation of the results into policy, making individuals less likely to take the time to respond.

The use of a both paper and digitised maps during the mapping process proved very effective, demonstrating the utility of this dual approach. Other mapping studies have annotated paper maps during interviews and then transformed them into digital form [36], [51], but direct annotation into the GIS is more resource efficient and removes the possibility of introducing error or bias during a later digitising process. Use of digitised maps also provides much greater flexibility, allowing the interviewer to carry multiple maps and work at different scales, depending on the type of fisher being interviewed. A pot fisher, for example, may work an area smaller than 5 km^2^ whereas a *Nephrops* fisher can be utilising the entire Irish Sea. Carrying around physical copies of sufficient maps, each with different resolutions to be appropriate for every fisher in each area is impractical, especially in the context of conducting interviews in small wheelhouses on boats. Digitised maps do not have this issue and can also be enhanced by adding or highlighting reference points, such as lighthouses. Furthermore, when using digital maps, built-in tools such as “zoom” and “pan” have been shown to facilitate the mapping process by revealing more scale-dependent detail [26]. Fishers also appreciated the ability to quickly and accurately measure distances on the digital map when they were drawing on their areas. Having some broad-scale paper maps available did prove useful, mainly as a comfortable starting point for those less technologically inclined, and the use of a combination seems to provide the best of both worlds.

SAPM facilitated the documentation of fishers’ actual access priorities, as opposed to interpreting their priorities from VMS-based mapping methods, and thus provides a fuller picture of which parts of the sea are most important to them. VMS alone maps only effort. Even when it is possible to incorporate log-book data with VMS data to estimate gross catch value of the different parts of the sea, this value is certainly not the only feature that makes an area important to fishers. Moreover, there are many boats that simply do not have VMS because they are too small. In Northern Ireland, over half the fleet would have their access priorities unaccounted for if planning were to use VMS-based maps alone.

Notwithstanding the limitations of VMS data, they remain vital for understanding the distribution of effort and are very important both for management and for informing the planning process. For example, they can be used to help predict the displacement impact of spatial restrictions. SAPM can be an important compliment to VMS based methods, when they are available, helping to compensate for their limitations. When they are not, it can be readily used as a standalone method.

This research also demonstrated how the SAPM method can be used to combine priorities across different types of fishery and to generate maps for the overall fleet, as well as for the separate fisheries. That SAPM can be used in any situation makes it suitable for enabling the direct comparison, and even combination, of the access priorities of multiple fleets, which otherwise might not have been possible due to differences in the associated data. Examples include: fleets that have VMS and fleets that do not, or fleets from different jurisdictions that access the same area but whose regulators have different fisheries data-capture and analysis methodologies.

Possibly the greatest advantage of SAPM over VMS is that it directly engages fishers in the process and allows them to contribute quantitative pre-planning data. The importance of stakeholder involvement is well recognised [20], [23]–[25], [33], [52] and is increasingly becoming a legislative requirement, incorporated into both national and international policy [23], [53]. Recent European Directives, such as the EU Public Participation Directive (2003/35/EC), have set new legal standards for stakeholder involvement, and almost every sustainability conference closes with a commitment to enhancing public participation [25].

Stakeholder involvement is sought because it has been shown to increase the quality and durability of environmental decisions, with the resultant plans better adapted to local socio-cultural and environmental conditions [23]–[25]. It can also reduce conflict and increase compliance [21], [33]. Indeed stakeholder participation and involvement have been widely identified as essential components of effective ecosystem management [54], [55], with differences in the extent and quality of participation being one of the key factors for variation in the quality of management approaches [20].

Stakeholder participation can also lead to the generation of additional data. Whilst undertaking SAPM, it would be relatively simple to gather other information, such as fishers’ opinions of management options, or local ecological knowledge, which can enhance understanding of the ecosystems being managed. Both can provide highly valuable input to the planning processes. Inclusion of stakeholder knowledge such as this can also help to integrate stakeholders into the planning process and to promote a sense of ownership [56]. Certainly here in Northern Ireland, fishers were more than happy to contribute, and discussions indicated that the inclusion of their knowledge and opinions into the planning process would enhance buy-in.

Stakeholder involvement is not without its opponents, with counterarguments suggesting that the participatory process may not in reality lead to better-quality plans, and that the high levels of participation may actually increase conflict by having disputing parties at the negotiating table [20]. There are concerns that it will frustrate the planner by slowing down the process and that the strength of the final agreement will be diluted to balance competing interests [20]. Protected area designs that accommodate social needs may also result in outcomes that do not adequately conserve ecosystems [57]. Yet evidence suggests that this need not be the case. The incorporation of stakeholders during the planning process can increase the likelihood of plan implementation and the presence of certain stakeholders, particularly industry, has been shown to significantly increase the quality of local ecosystem plans [20]. Likewise, research shows that the use of fisheries data in MPA selection can reduce the negative impacts on the industry whilst still meeting conservation goals [58], [59], and that incorporation of socio-economic considerations into systematic conservation planning can improve overall cost effectiveness [60]. It has also been frequently demonstrated that when fishers are involved in the research and decision-making process, management guidelines are more likely to be effective [26], [61], [62].

Another concern might be the credibility of stakeholder involvement, questioned on the basis that many may not have sufficient expertise to meaningfully engage in what are often technical debates [63]. However, in this case it is actually the fishers that are the experts, often with decades of fishing experience. This acumen of fishers is increasingly recognised, with more and more studies documenting their extensive ecological knowledge [52], [64], [65] and surely who better to indicate which parts of the sea are most important to fishers than the fishers themselves?

With stakeholder consultation becoming more and more integrated into governance, the issue of consultation fatigue can also arise: when stakeholders are asked to take part in multiple or multi-stage participatory processes in which they perceive their involvement gains them little reward or capacity to influence the decisions being made [66]. Use of SAPM can help prevent that. It collects data in a systematic way, one of the recommendations for overcoming barriers to stakeholder participation [67]. Participation in SAPM is also of low cost to fishers and the method is readily explained. Resultant data are baseline preplanning data, so they can be used for multiple different planning projects, helping to avoid excessive or repeated demands on fishers’ time. The maps generated can easily be used to demonstrate the results of the research and how that can feed into the planning process. They can also be readily incorporated into conservation planning software, such as MARXAN, where their influence on selecting potential MPA site can be illustrated.

As mentioned earlier, the cost of participation at centrally based stakeholder events can be a barrier to participation to fishers. Participation tends to be higher amongst those with more extreme positions and amongst those who are more dominant, influential and financially powerful within a community [68], so those fishers who do attend central events are unlikely to be truly representative of the fleet as a whole. By actually going to the fishers, SAPM can help to ensure a more diverse range of fishers is involved in the planning process. It also allows much larger numbers to partake: whilst it would be impractical for DOE to have 100 fishers around a planning table, SAPM allowed those fishers to contribute in a way that made them feel part of that process.

Other studies have looked at incorporating fishers’ priorities into marine planning. In particular, a series of studies incorporating Californian fishers’ knowledge and priorities into MPA selection has been conducted by Klein [58], [69] and Scholz [31], [32], [70]. Fishers were asked to map their fishing grounds and to indicate relative importance [70], in a similar way to SAPM. The study area was then divided into 1381 planning units and the fishers data was used to calculate the relative importance of those planning units, across all fisheries and within a fishery [58]. Surrogate layers for relative fishing effort for each fishery were produced, and by summing the layers for the different fisheries they were able to develop an index of relative effort across all fisheries. In this method, each fishery receives equal weighting, such that a fishery that supported 10 fishers would have the same input into the final index as one that supported 1000. No weighting is applied to the individual fishers’ areas either, so that results from a skipper whose vessel supported 10 crew would have the same weight as result from a skippers who vessel supported two.

This approach does increase the likelihood that all fisheries would be affected equally, which in this instance was a specific goal of the stakeholders (Klein, pers.comm). However, it is also often very desirable to minimise total negative impact on the fishing community and protect the greatest number of livelihoods. In later studies, Klein *et al* [69] and Scholz *et al* [32] incorporated a weighting system. They weighted the area(s) each fisher indicated by their vessel’s revenue, which means that those fishers with vessels that caught more and made more profit had proportionally more input. This is similar to SAPM, where responses are weighted by the number of crew, so that those vessels with more crew have more total priority. Weighting by vessel revenue enabled Klein *et al* [69] and Scholz *et al* [32] to produce layers that showed the monetary value, in US dollars, of a given planning unit to an individual fisher. They summed those individual layers to create cost layers, which they used in Marxan to develop MPA planning options that minimised the negative impacts of spatial restrictions on the fishing industry.

The main difference between previous approaches and SAPM is that SAPM can allow an assessment of both relative and total access priority, and the production of surrogate cost layers, without the need for additional fisheries data. This is because SAPM uses information derived from interview (crew numbers), rather than revenue or landings, to weight the responses. Like the cost layer(s) created in both Klein *et al* [69] and Scholz *et al* [32], the SAPM generated surrogate cost layer(s) can be used in Marxan to minimise total negative impact of MPA designation on the fishing community. If the goal was to increase the likelihood that all fisheries are affected equally, like Klein *et al* [58], SAPM can also be used to map and assess relative priority, by dividing the SAP km^−2^ by the total SAP of a fishery and/or a fleet. Moreover, SAPM can also be used to assess the equality of spatial restrictions by analysing the SAPs of different port-gear-length combinations and comparing how much a given restriction or set of restrictions affects each group. When it is possible to access other data, such as revenue data, SAPM can be easily modified to incorporate it, see below for some possible adaptations to the method.

Another major difference is that SAPM specifically takes account of the different characteristics of the vessels (length/port/gear) when weighting and scaling responses. This is important because size of a vessel and the port from which it operates are both likely to have an impact on its preferred grounds, even when fishing for the same target species. Furthermore, the previous studies did not ascertain whether their samples (the fishers they interviewed) were representative of the fleet. Indeed, Scholz *et al* [32] state that to reach their sampling goal as efficiently and cheaply as possible they targeted fishers with the largest catches [32]. Whilst understandable, this suggests that, within a given fishery, the fishers with the smallest vessels had a lower chance of being interviewed. Smaller vessels tend to be more restricted in terms of fishing grounds and may have totally different spatial access priorities than larger vessels. By requiring a representative sample of the fleet SAPM helps to ensure all fishers are included, essential when aspiring to equitable stakeholder involvement.

Use of SAPM does not remove the need for subsequent engagement with the fishing community, nor will it remove all the potential conflict associated with MSP and MPA designation. It is a beginning, not an end: providing data to feed into a planning process and allowing a planner to assess the impact of different potential plans on fishers. Its use should improve the transparency of the planning process, enhance buy-in and increase trust.

### Possible Adaptations to the Method

The weighting system used in SAPM is highly flexible, allowing for the incorporation of livelihoods or economic values beyond the vessels’ crews. In Northern Ireland, for example, fish processors employ an estimated 500 full-time staff [43]. To incorporate these into the analysis, those 500 would be first separated into their associated fisheries. Then, based on the percentage of fishers within each length-gear-port combination for that fishery, they would be divided between each combination and added to the estimated number of crew. Doing this would proportionally increase the priority of fisheries with associated local processing, such as *Nephrops*, and cause a relative decrease in those without, reflecting the higher numbers of livelihoods that depend on those sectors. Similarly, if other data, such as landings, are available, the weighting system could also be modified accordingly. The total economic value of landings for a given fishery could be divided by the number of fishers in that fishery to obtain a value per fisher, which would then be multiplied by the weighting obtained from the standard method for each length/gear/port combination.

### Future Research

This research did not aim to investigate the fishers’ motivations for selecting different areas or for assigning priorities. Nor were they restricted to assigning priorities for a specific time span. It would be informative to expand future SAPM projects to include a debriefing session, where researchers explore with fishers their rationale and motivation for selecting areas and assigning priorities. An improved understanding of what makes areas important to fishers may help with predicting how they respond to spatial and temporal restrictions. Future SAPM projects could also incorporate the gathering of local ecological and historical knowledge, including: mapping where the fishers historically accessed resources and how their SAPs have changed over time, discussing when and why they changed, and comparing answers to legislative, ecological and climatic trajectories.

### Conclusions

Spatial management can have many benefits for conservation and fisheries alike, but in order to realise them stakeholders need to be engaged in a strategic manner from an early stage. SAPM offers a tool that not only gathers quantitative data on fishers’ access priorities, but also facilitates stakeholder engagement and increases the opportunity for dialogue between fishers and managers. The resultant maps provide a transparent, easy to demonstrate way of incorporating fishers’ priorities, especially when combined with spatial planning software such as MARXAN. The use of SAPM should facilitate strategic conservation planning, maximising benefits whilst both minimising negative impacts on stakeholders and helping to ensure those impacts are spread equitably. We hope that the inclusive planning that SAPM encourages will lead to more successful solutions to the inevitable conflicts of interests that conservation planning encounters.

## Supporting Information

File S1 Pre-interview Information sheet. Pre-interview information sheet provided to fishers prior to participation in a survey targeting skippers and owners of commercial fishing vessels registered in Northern Ireland. After the potential participant had read the information sheet, the content was discussed to ensure understanding and obtain verbal consent, before beginning the interview. Research was completed during 2012.
(PDF)

File S2 Questionnaire. Questionnaire used to guide semi-structured interviews of skippers and owners of Northern Ireland registered commercial fishing vessels. Interviews were preceded with pre-interview information sheets. 103 interviews were conducted in total, during 2012. The responses from some of these questions were used to develop Spatial Access Priority Maps, this study, whilst others were used to answer research questions explored in subsequent studies.
(PDF)
