# Supplementary material for: Spatial Access Priority Mapping (SAPM) with Fishers: A Quantitative GIS Method for Participatory Planning
Source: PLoS One. 2013 Jul 16;8(7):e68424. doi: 10.1371/journal.pone.0068424 (PMC3713016; doi:10.1371/journal.pone.0068424)
Supplement: File S1 — Pre-interview Information sheet. Pre-interview information sheet provided to fishers prior to participation in a survey targeting skippers and owners of commercial fishing vessels registered in Northern Ireland. After the potential participant had read the information sheet, the content was discussed to ensure understanding and obtain verbal consent, before beginning the interview. Research was completed during 2012. (PDF) [file pone.0068424.s001.pdf]

Information Sheet

Before the interview begins the following information will be discussed with you. It outlines the purpose of the research and how the information you provide will be analysed and use. Please ask any questions and remember all your individual information will be confidential and anonymised.

The Diverse Seas Project is an independent PhD research project being conducted by myself, Katherine Yates, from the University of Ulster. The overall project focuses on strategic marine planning and you have been invited here today to be part of research to determine the spatial access priorities of Northern Irelands fishing fleet.

Today's interview should last between 30 minutes and an hour, depending on how many questions you have and how much information you are able to provide. There will be a few very short questions to start, such as the name of your boat and the type of licence(s) you have. Then we will move on to mapping the areas of the sea that are most important to you as a fisher.

On top of admiralty charts, we will draw on your priority areas. You can identify as many or as few areas as you like and as much overall area as you wish. However the greater the total area the lower the priority each unit of area will have, thus there is a great benefit in being specific. You can choose to allocated different areas different levels of priority, for example: this area is twice as important as the others are. You will also be asked about the impact of changing fuel prices on the areas that are important to you. You will asked if there are any areas you think should be MCZ's and if there are any areas of particular biological interest.

Your data will be combined with the other fishers interviewed to create an access priority maps, showing which areas of the sea are most important to the Northern Ireland fishing community. The research, including these maps, will be presented to the various government departments that are involved in marine planning and management. It is hoped that this will enabled the access needs of the fishing community to be considered right at the start of the marine spatial planning and MCZ planning processes.

All information is confidential and anonymised. No individual responses will be available to anyone other than me. The report and access priority maps will be available to everyone. All fishers and Fish Producer Associations will have the opportunity to review and comment on a draft copy of the report and maps before they are finalised and published. All comments will be included in the report appendix.

University of Ulster, Coleraine Katherine Yates yates-k1@email.ulster.ac.uk
