# Supplementary material for: Spatial Access Priority Mapping (SAPM) with Fishers: A Quantitative GIS Method for Participatory Planning
Source: PLoS One. 2013 Jul 16;8(7):e68424. doi: 10.1371/journal.pone.0068424 (PMC3713016; doi:10.1371/journal.pone.0068424)
Supplement: File S2 — Questionnaire. Questionnaire used to guide semi-structured interviews of skippers and owners of Northern Ireland registered commercial fishing vessels. Interviews were preceded with pre-interview information sheets. 103 interviews were conducted in total, during 2012. The responses from some of these questions were used to develop Spatial Access Priority Maps, this study, whilst others were used to answer research questions explored in subsequent studies. (PDF) [file pone.0068424.s002.pdf]

Prelim Questions

1) What is the name of your boat(s)?

2) How many people work on it? full time, part time (hours?), seasonal (when?)

3) From which port is it based?

4) What age were you when started working in the fishing industry in Northern Ireland as a professional fisher?

5) What age were you on your last birthday?

6) Is fishing your full time job? Yes/No
   i. If no, during 2011 how many hours a week did you usually spend working as a fisher?
      1. Less than 10
      2. 10-20
      3. 21-30

7) Which fisheries do you have or have you ever had a commercial licence for?

Fishery	Currently	Never	In the past
White fish			
Nephrops			
Scallop			
Potting			
Herring			
Mackerel			
Other, please specify			

8) If you held licences in the past:
   i. What were your main reasons for leaving the fishery?
   ii. In what year did you last have a licence for that fishery/those fisheries?
   iii. Do you still have the gear for that fishery/those fisheries?
   iv. Is there anything that would make you consider returning to that fishery?

9) If you have licences for multiple fisheries:
   - For each of the last three years, how much of your time did you focus on each fishery?

Fishery	2011	2010	2009

Spatial Questions

1) Highlight on the map the parts of the sea that are most important for you to have access to. You can highlight as many different areas as you like and as much total area as you wish. However, the more area you highlight the less weight each unit of area will have, thus there is a great benefit to being specific. You can also assign each area you highlight different levels of importance, for example this area is twice as important as that one or I do 80% of my fishing here.

2) Between 2001 and 2010 the price of red diesel has more than doubled.
   a. Has this impacted where and how you fish and which areas are most important to you have access to? Please indicate on the map.

   b. If fuel prices were to return to 2001 prices, would this impact where you fish and which areas are most important for you to have access to? If so please indicate on the map

3) Are there any areas that you think should be protected in the future? If so where, why and from what activity/activities?

4) Can you indicate any areas of particular biological or fishing interest?
   Prompts: High diversity areas, spawning grounds, nursery grounds, historic fishing grounds, areas where you have witnessed change, areas where rarer species are more abundant

5) Are there any comments you would like to add?
   Prompts: General state of the industry, issues, concerns, management suggestions

University of Ulster, Coleraine Katherine Yates yates-k1@email.ulster.ac.uk
